# Mapping neuroimaging research related to post-traumatic headache after traumatic brain injury: a multi-database bibliometric analysis

**DOI:** 10.3389/fnins.2026.1923958

**Published:** 2026-09-09

**Authors:** Ruiting Zhu, Lu Zhang, Xingpu Quan, Jiawen Hu, Siyuan Chen, Yanan Zhu, Feng Liu, Mangmang Bai, Jingling Qiang, Xuan Niu, Ming Zhang, Dongbo Li

**Affiliations:** 1 School of Future Technology, Xi’an Jiaotong University, Xi'an, Shaanxi, China; 2 Department of Neurosurgery, Ankang Central Hospital, Ankang, Shaanxi, China; 3 Department of Medical Imaging, The First Affiliated Hospital of Xi’an Jiaotong University, Xi’an, Shaanxi, China; 4 Medical Imaging Centre, Ankang Central Hospital, Ankang, Shaanxi, China; 5 Department of Neurosurgery, The Affiliated Hospital of Yan’an University, Yan’an, Shaanxi, China

**Keywords:** bibliometrics, neuroimaging, post-traumatic headache, traumatic brain injury, visualization analysis

## Abstract

**Background:**

Post-traumatic headache (PTH) is one of the most common and disabling sequelae of traumatic brain injury (TBI). Neuroimaging has been increasingly used to evaluate acute injury, identify structural and functional abnormalities, and explore the mechanisms underlying persistent headache after TBI. However, the global development and research trends in this field remain unclear. This study aimed to map the knowledge structure and emerging trends of neuroimaging research related to PTH after TBI.

**Methods:**

A comprehensive literature search was conducted in the Web of Science Core Collection (WoSCC), Scopus, and PubMed from database inception to December 31, 2025. WoSCC and Scopus were used as the principal bibliometric dataset, while PubMed was used for supplementary validation. Bibliometrix, VOSviewer, and CiteSpace were used to analyze publication trends, countries, institutions, journals, authors, cited references, keyword co-occurrence, thematic evolution, and trend topics.

**Results:**

After merging WoSCC and Scopus records, 2,519 documents from 938 sources were included in the main analysis, and 539 PubMed records were used for supplementary validation. Annual publication output showed an overall upward trend over the study period. The United States and Harvard University were the most productive country and institution, respectively, whereas the *Journal of Neurotrauma* and Schwedt TJ showed the highest source and author influence. Within the broader literature encompassed by this search, highly cited publications included studies on CT-based acute brain injury assessment, pediatric risk stratification, PTH diagnostic classification, and post-concussion symptoms. Keyword and thematic analyses revealed a gradual transition from acute imaging evaluation toward advanced neuroimaging, outcome assessment, prognosis, and rehabilitation.

**Conclusion:**

Within this broad interdisciplinary multi-database bibliometric dataset linking TBI, PTH and neuroimaging, we identified a long-standing foundation in acute TBI imaging and related symptom assessment, alongside an increasingly visible research trajectory focused on advanced neuroimaging of persistent symptoms, mechanisms, prognosis, and rehabilitation-oriented management.

## Introduction

Traumatic brain injury (TBI) is brain damage caused by physical blows to the head from external forces, including traffic accidents, falls, bumps, or assaults (Maas et al., 2022). It remains a major global public health burden, with approximately 69 million new cases estimated to occur annually worldwide (Maas et al., 2022; Dewan et al., 2019). Beyond the acute neurological injury, TBI is frequently followed by a broad range of sequelae, such as headache, fatigue, disturbance, depression, and anxiety (Ashina et al., 2019; Polinder et al., 2018). These symptoms may persist beyond the acute stage and substantially impair patients’ physical, psychological, and social functioning (Eagle et al., 2025).

Post-traumatic headache (PTH) is a common sequela of TBI, although reported incidence varies according to injury severity, study setting, case definition, and follow-up duration. In a prospective cohort of mild TBI patients, Lucas et al. reported a 91% cumulative incidence of new or worsened headache over the first year after injury (Lucas et al., 2014). According to the International Classification of Headache Disorders, 3rd edition (ICHD-3), PTH attributed to TBI requires temporal association with the injury, with headache occurring within 7 days after injury, within 7 days after regaining consciousness, or within 7 days after discontinuation of medications that impair the ability to sense or report headache. Acute PTH refers to traumatic headache that has resolved or persists within 3 months of onset, whereas persistent PTH refers to headache that lasts for more than 3 months after onset [Headache Classification Committee of the International Headache Society (IHS), 2018]. PTH may persist after the acute phase in a substantial proportion of patients. In a prospective multicenter TRACK-TBI cohort, nearly 30% of patients with acute PTH continued to report persistent PTH at 12 months after mild TBI, emphasizing its potential long-term clinical burden (Ashina et al., 2023).

However, the manifestations of PTH are often heterogeneous and may resemble migraine or tension-type headache phenotypes (Lucas et al., 2014). Because PTH lacks highly specific clinical features, its diagnosis and evaluation remain challenging, especially in patients with mild TBI who experience persistent symptoms despite no abnormalities on conventional imaging (Christensen et al., 2025a; Schwedt, 2021). Therefore, early identification, systematic evaluation, and mechanistic exploration of PTH after TBI are important for improving clinical management and long-term outcomes.

Neuroimaging has played an increasingly important role in TBI research. Conventional imaging modalities, particularly computed tomography (CT), are widely used in the acute setting to identify clinically significant intracranial lesions and guide immediate management (Rau et al., 2018; Haydel et al., 2000). In contrast, advanced neuroimaging techniques, including diffusion tensor imaging (DTI), resting-state functional magnetic resonance imaging (rs-fMRI), magnetic resonance spectroscopy (MRS), and positron emission tomography (PET), provide opportunities to detect subtle structural, functional, and metabolic alterations after TBI. PTH attributed to TBI is fundamentally a clinical diagnosis based on the temporal relationship between headache onset and TBI together with the clinical presentation. Further, previous studies have reported structural, white-matter, and functional network alterations associated with PTH (Ofoghi et al., 2020). DTI examinations have shown abnormal white matter tract integrity in regions such as the corpus callosum and fornix (Kim et al., 2024), whereas functional imaging studies have suggested altered static and dynamic brain network connectivity in patients with PTH after mild TBI (McGeown et al., 2026; Li et al., 2021). Thus, when clinically indicated, neuroimaging may help identify traumatic structural abnormalities and characterize structural, functional, metabolic, and network-level alterations associated with PTH and persistent post-traumatic symptoms.

Despite these advances, neuroimaging research related to PTH after TBI remains scattered across several partially overlapping fields, including neurotrauma, headache medicine, neuroradiology, cognitive neuroscience, and rehabilitation (Ofoghi et al., 2020; McGeown et al., 2026). This field also encompasses different clinical purposes, from acute lesion detection and imaging triage to mechanism-oriented advanced imaging and long-term outcome prediction. This structure makes it difficult to obtain a comprehensive view of its development trajectory, knowledge base, research hotspots, and emerging frontiers.

Bibliometric analysis provides a quantitative and visual approach for mapping the development of a research field (Donthu et al., 2021). Unlike conventional systematic reviews that typically explore specific research questions through detailed comprehensive analysis of a relatively focused evidence set, bibliometric analysis is particularly suited to broad and interdisciplinary research fields, aiming to describe their knowledge structures, collaboration networks, knowledge foundations, research hotspots, and emerging topics (Zupic and Čater, 2015). Bibliometric methods have been applied to TBI-related research to explore the development trend of TBI rehabilitation (Mojgani et al., 2022), the current research status of post-TBI dementia (Sang et al., 2023), and leading-edge hotspots in the relationship between TBI and gut microbiota (Du et al., 2023). However, no study has systematically mapped the global research landscape of neuroimaging in PTH after TBI. Notably, this research topic has evolved across several interrelated fields, including neurotrauma, emergency medicine, neurosurgery, headache medicine, neuroradiology, and rehabilitation etc. Therefore, the present study aimed to analyze the current status, knowledge structure, and emerging trends of this field within this interrelated clinical and imaging context. By integrating visual knowledge mapping across multiple databases and software platforms, this study seeks to clarify how the field has developed and to provide references for future clinically oriented neuroimaging studies.

## Methods

### Data sources and search strategy

A comprehensive literature search was conducted in the Web of Science Core Collection (WoSCC), Scopus, and PubMed databases. WoSCC and Scopus were used as the principal data sources for bibliometric analysis because they provide relatively complete bibliographic and citation metadata. PubMed was used as a supplementary database for cross-database validation because of its strong biomedical coverage. Searches were performed on April 1, 2026, and records published from database inception to December 31, 2025 were included. All database searches were completed on the same day to reduce potential bias caused by database updates.

Search strategy was constructed around three conceptual blocks: traumatic brain injury, post-traumatic headache, and neuroimaging. Search terms related to TBI were designed to cover TBI across various severity levels, including mild TBI literature indexed or described using concussion terminology. The WoSCC Topic search was used as the primary search framework and was then adapted to the syntax and indexing structure of Scopus and PubMed. In Scopus, the corresponding terms were searched using TITLE-ABS-KEY. In PubMed, MeSH terms and Title/Abstract terms were combined where applicable to account for its controlled vocabulary and biomedical indexing system.

WoSCC search strategy was as follows: TS = (“Brain Injuries, Traumatic” OR “Brain Injury, Traumatic” OR “Traumatic Brain Injuries” OR “Trauma*, Brain” OR “Brain Trauma*” OR “TBI*” OR “Encephalopathy, Traumatic” OR “Encephalopathies, Traumatic” OR “Traumatic Encephalopathies” OR “Injury, Brain, Traumatic” OR “Traumatic Encephalopathy” OR “Traumatic Brain Injury” OR “Cerebral trauma” OR “Post-traumatic” OR “Post-concussion” OR “Concussion*” OR “Concussive” OR “Commotio Cerebri”) AND TS = (“Headache*” OR “Secondary Headache” OR “Post-Traumatic Headache” OR “Head Pain*” OR “Pain*, Head” OR “Cephalalgia*” OR “Cranial Pain*” OR “Cephalgia*” OR “Hemicrania”) AND TS = (“imaging” OR “neuroimaging” OR “neuroradiology” OR “computed tomography” OR “CT” OR “MRI” OR “DKI” OR “DWI” OR “DTI” OR “PWI” OR “arterial spin labelling” OR “ASL” OR “magnetic resonance spectroscopy” OR “MRS” OR “positron emission tomography” OR “PET” OR “single photon emission computed tomography” OR “SPECT” OR “network” OR “connect*” OR “ultrasound” OR “doppler”).

Equivalent searches were performed in Scopus and PubMed while preserving the same conceptual framework, keyword combinations, Boolean operators, time span, and screening criteria. The specific search strategies are shown in Supplementary Tables S1, S2.

### Inclusion and exclusion criteria

Studies met the following criteria: (1) original research publications or reviews; (2) studies related to PTH after TBI; (3) studies involving neuroimaging, imaging evaluation, or imaging-related mechanisms; and (4) studies published in English.

Exclusion criteria were as follows: (1) non-English publications; (2) meeting abstracts, editorials, letters, comments, news items, and book chapters; (3) retracted publications; (4) records with incomplete bibliographic information; and (5) studies clearly irrelevant after manual screening.

### Data cleaning and standardization

**Figure 1:**
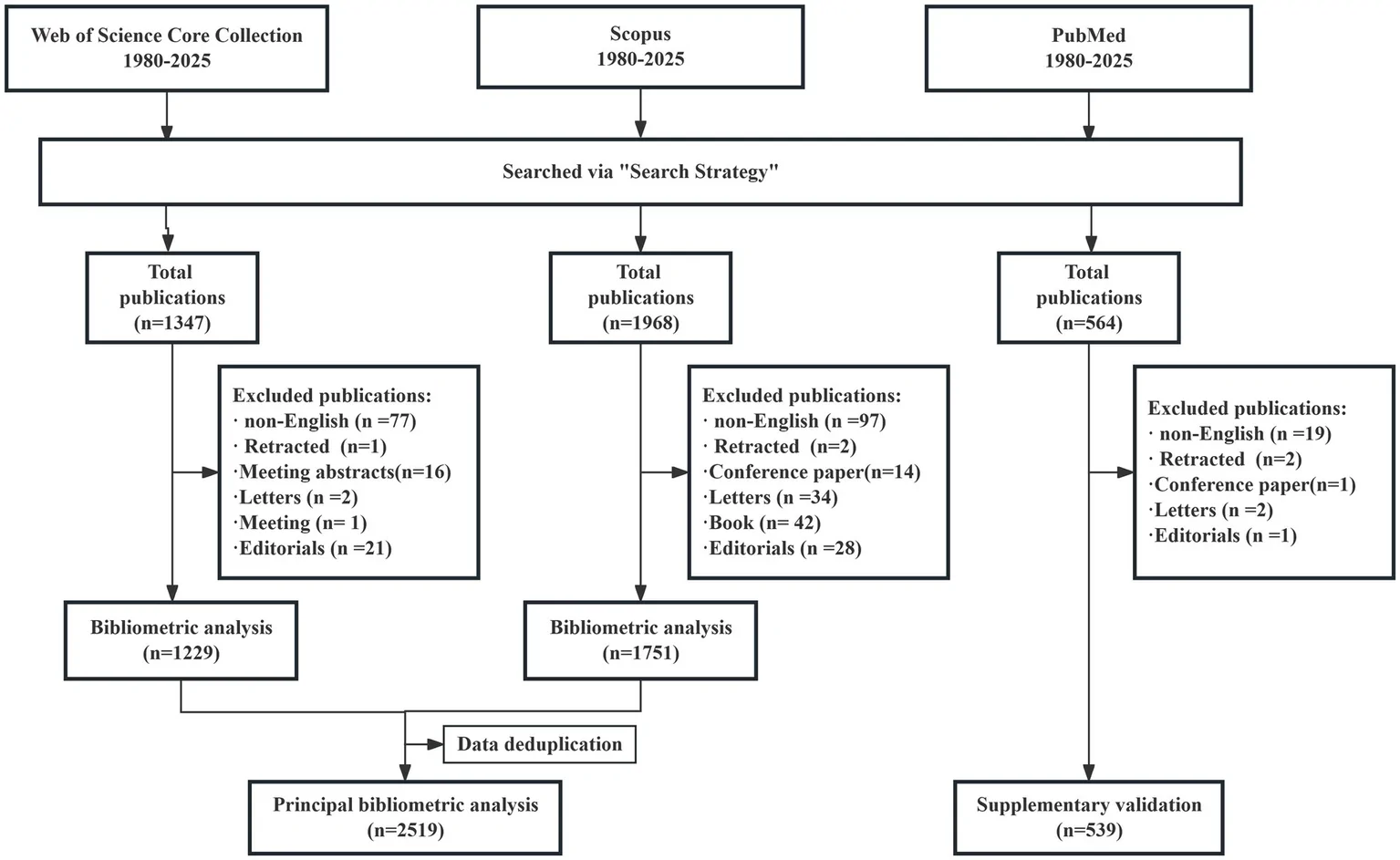
Flow diagram of the study selection process.

After retrieval, WoSCC and Scopus records were merged in bibliometrix, and duplicate detection was performed using a hierarchical procedure. DOI was used as the primary identifier when available. For records without a DOI, titles were compared after accounting for small differences in capitalization, punctuation, spacing, and database-specific formatting. Subsequently, potential duplicate records were cross-checked using first author, publication year, and journal/source information. Ambiguous records were manually reviewed and were removed only when duplication could be confirmed. This process was independently conducted by two researchers, with any disagreements resolved through discussion. Assessment of inclusion eligibility was based on whether there were substantive associations between research content and neuroimaging of PTH after TBI. Studies were included in this bibliometric analysis if they provided substantive clinical, imaging or interdisciplinary context directly relevant to the development of this field and met the inclusion and exclusion criteria. WoSCC and Scopus records were then merged to construct the main bibliometric dataset. PubMed records were retained for supplementary validation. The screening and selection process is shown in Figure 1. The document type labels from each database were harmonized according to substantive publication types. Eligible full-length publications reporting original research were classified as original research, while review publications were classified as reviews.

Before analysis, author, institutional affiliations, country/region, and keywords were standardized as far as possible to reduce the influence of spelling variants, abbreviations, capitalization differences, and inconsistent database indexing. Obvious institutional variants were merged when they referred to the same affiliation entity. For example, variants related to Harvard University were standardized under Harvard University where appropriate. We standardized institution names manually according to bibliometric data-cleaning practice (Montazeri et al., 2023).

To further examine the relevance of the included publications to neuroimaging research related to PTH after TBI, 200 records were randomly selected from the main bibliometric dataset and manually re-evaluated. The records were categorized into four categories based on research focus and substantive relevance. Detailed definitions of each category are provided in Supplementary Table S3. Two reviewers independently classified the sampled records, and any disagreements were resolved through discussion.

### Bibliometric analysis and visualization

VOSviewer (version 1.6.20) (van Eck and Waltman, 2010), CiteSpace (version 7.0.0) (Chen, 2006), the bibliometrix R package with its Biblioshiny web interface (version 5.0) (Aria and Cuccurullo, 2017), and Microsoft Excel 2021 were used for bibliometric analysis and visualization. WoSCC and Scopus datasets were merged in Biblioshiny to construct the main bibliometric dataset. Bibliometrix was used to perform descriptive bibliometric analysis, including annual scientific production, source analysis, country/region and institutional output, author analysis, citation analysis, thematic evolution, and trend topics. The annual growth rate was calculated by bibliometrix as the compound annual growth rate of publication output between the first and last publication years, $[{\left(P_{\text{last}}/P_{\text{first}}\right)}^{Y_{\text{last}}-Y_{\text{first}}}-1]\times 100\%$ , where $P_{\text{first}}$ and $P_{\text{last}}$ represent the numbers of publications in the first and last publication years, respectively, and $Y_{\text{first}}$ and $Y_{\text{last}}$ represent the corresponding years. VOSviewer was used to construct collaboration and keyword co-occurrence networks. CiteSpace was used to identify keywords with strong citation bursts and to detect emerging research frontiers. Microsoft Excel was used to generate line graphs and summary tables.

Source and author influence was assessed using H-index, G-index, M-index, publication counts, and citation counts calculated from publications included in the analyzed bibliometric corpus (Hirsch, 2005; Egghe, 2006). These metrics reflect topic-specific influence within this dataset. Institutional contribution was evaluated based on publication output after affiliation name standardization. Local citations (LC) were defined as citations received from other documents within the analyzed bibliometric corpus, while global citations (GC) represented the total citation count of that document in the source database. LC reflects field-specific influence, whereas GC reflects broader citation impact.

### Document-type sensitivity analysis

To evaluate the influence of original research publications and reviews on principal bibliometric results, a sensitivity analysis was conducted for the included original research publications after excluding reviews. Pearson’s correlation coefficient was used to compare annual publication trends between the complete dataset and subset containing only original research publications. Jaccard similarity and Spearman’s rank correlation coefficients were used to evaluate the consistency of top 15 journals and keywords. Additionally, citation distributions between original research publications and reviews were compared using the mean and median total citation counts.

### Multi-database validation

To assess the robustness of the bibliometric findings, annual publication trends, standardized top keywords, and core journals were compared across WoSCC, Scopus, and PubMed. Pearson’s correlation coefficient was used to evaluate the consistency of annual publication trends across databases. Jaccard similarity was used to measure the overlap of standardized top keywords and core journals. Spearman’s rank correlation coefficient was used to assess the consistency of keyword and journal rankings across databases. This validation strategy was designed to distinguish robust macro-level trends from findings that may be more sensitive to database coverage, indexing practices, and source inclusion patterns.

## Results

### Literature selection and dataset characteristics

Finally, WoSCC included 1,229 documents from 504 sources, and Scopus included 1,751 documents from 707 sources. After merging WoSCC and Scopus and removing duplicates, 2,519 documents from 938 sources were retained in the principal bibliometric analysis, comprising 2,008 original research publications (79.7%) and 511 reviews (20.3%). In addition, 539 PubMed records from 273 sources were included for supplementary validation (Figure 1).

The merged dataset had an annual growth rate of 1.55% and an average of 24.01 citations per document. It contained 11,917 Keywords Plus, 5,031 Author Keywords, and 12,274 authors. The average number of co-authors per document was 5.62, and the international co-authorship rate was 6.58% (Table 1).

**Table 1:** Overview of PTH after TBI in different databases.

Description	WoSCC	Scopus	WoSCC + Scopus	PubMed
Main information				
Timespan	1980:2025	1980:2025	1980:2025	1980:2025
Sources (journals, etc.)	504	707	938	273
Documents	1,229	1751	2,519	539
Annual growth rate, %	3.53	10.23	1.55	8.47
Average citations per document	28.13	23.49	24.01	NA
Document contents				
Keywords plus	2,531	10,919	11,917	823
Author keywords	2,816	3,566	5,031	1,070
MeSH terms	NA	NA	NA	823
Authors				
Total authors	6,318	8,603	12,274	2,724
Authors of single-authored documents	60	113	158	25
Authors cooperation				
Single-authored documents	62	130	173	26
Co-authors per document	5.74	5.80	5.62	6.28
International co-authorship, %	13.43	15.53	6.58	12.55

TBI, traumatic brain injury. PTH, post-traumatic headache. NA, not available.

In relevance validation of 200 randomly selected records, 17 studies (8.5%) were classified as neuroimaging studies on PTH after TBI, 20 (10.0%) were classified as TBI neuroimaging studies incorporating PTH assessment, 113 records (56.5%) demonstrated a significant contextual relevance to the field, while 50 (25.0%) belonged to broader interdisciplinary studies but had a clear and meaningful connection to this field (Supplementary Table S3). These results indicate that this bibliometric dataset covers a variety of studies related to neuroimaging of PTH after TBI, ranging from specialized research on this topic to broader clinical and imaging studies that contribute to the advancement of this interdisciplinary field.

### Annual publication trends

**Figure 2:**
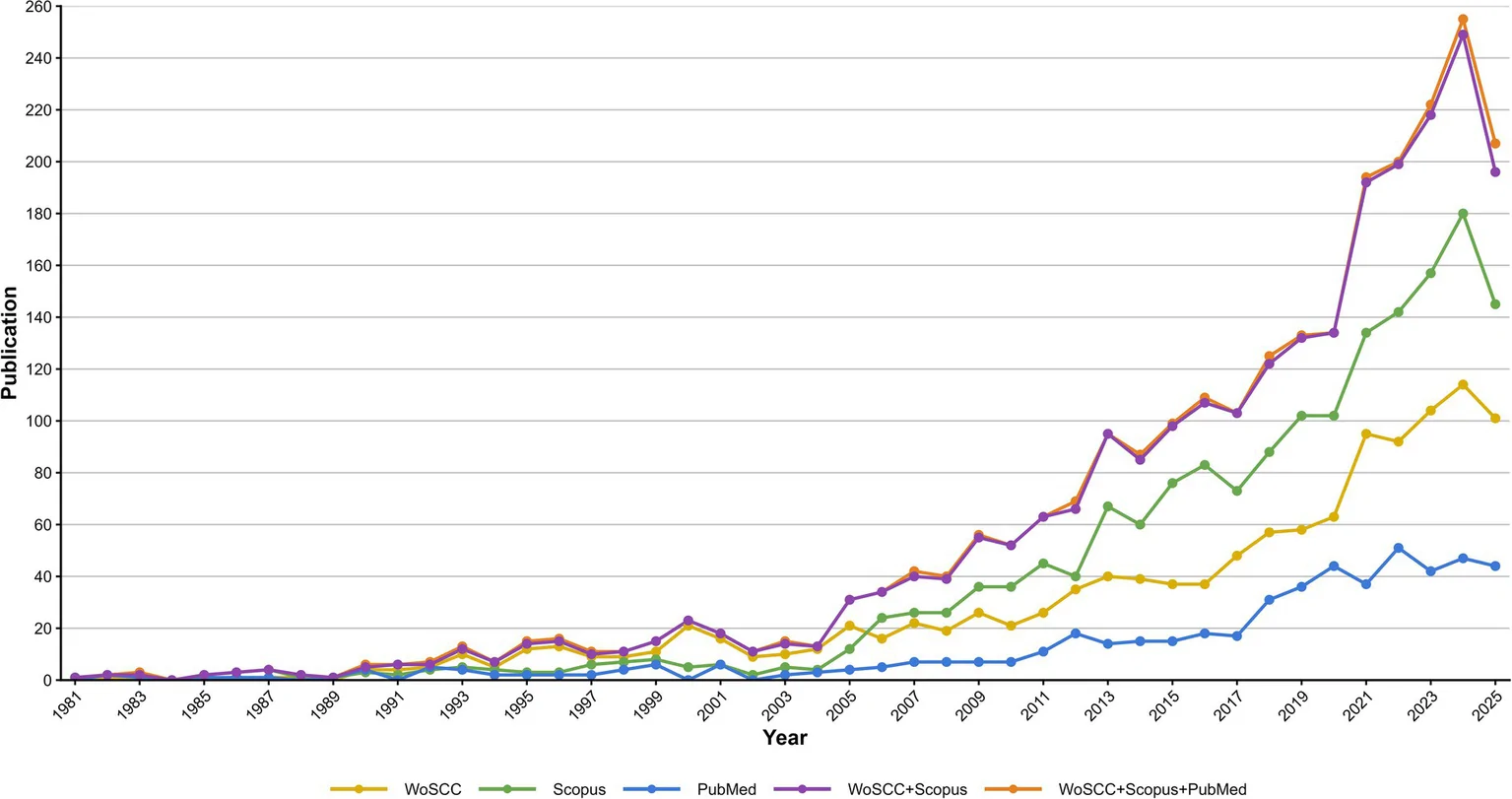
Annual scientific production across databases.

As shown in Figure 2, annual scientific production showed an overall upward trend across the three databases. Publication output was relatively low during the early period and generally increased over time, with comparatively higher annual output from 2021 to 2025.

### Source analysis

**Figure 3:**
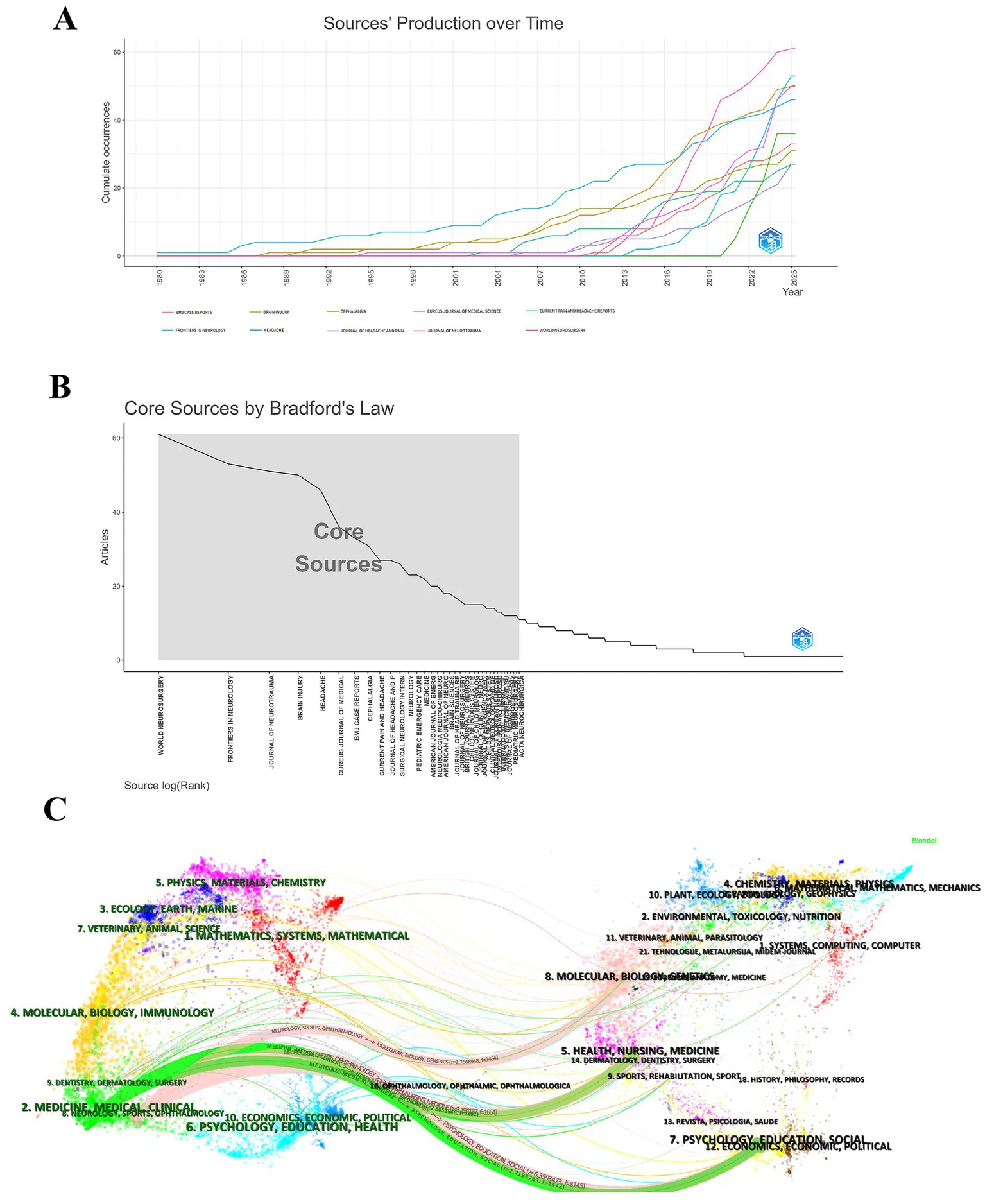
Visualization of journal sources. **(A)** Annual production trends of the top 10 journals in terms of publication quantity. **(B)** Core journals determined according to Bradford’s law. **(C)** Dual map overlay illustrating the disciplinary distribution and citation pathways of journals.

The source analysis showed a relatively concentrated journal structure (Figure 3). According to Bradford’s law, a limited number of core journals accounted for a substantial proportion of publications in this field. Among the top 10 journals ranked by H-index, *Journal of Neurotrauma* ranked first, with an H-index of 22, 51 publications, and 2,147 citations. It was followed by *Headache* (H-index = 20, 46 publications, 1,037 citations), *Cephalalgia* (H-index = 17, 31 publications, 1,017 citations), and *Brain Injury* (H-index = 16, 50 publications, 1,308 citations) (Supplementary Table S4). Although *World Neurosurgery* had the largest number of publications within the top 10 journals, with 61 articles, its H-index was lower than that of several leading journals in neurotrauma and headache medicine. Overall, the core sources were mainly distributed across neurotrauma, headache medicine, neurology, and neuroradiology-related journals.

### Analysis of countries/regions and institutions

**Figure 4:**
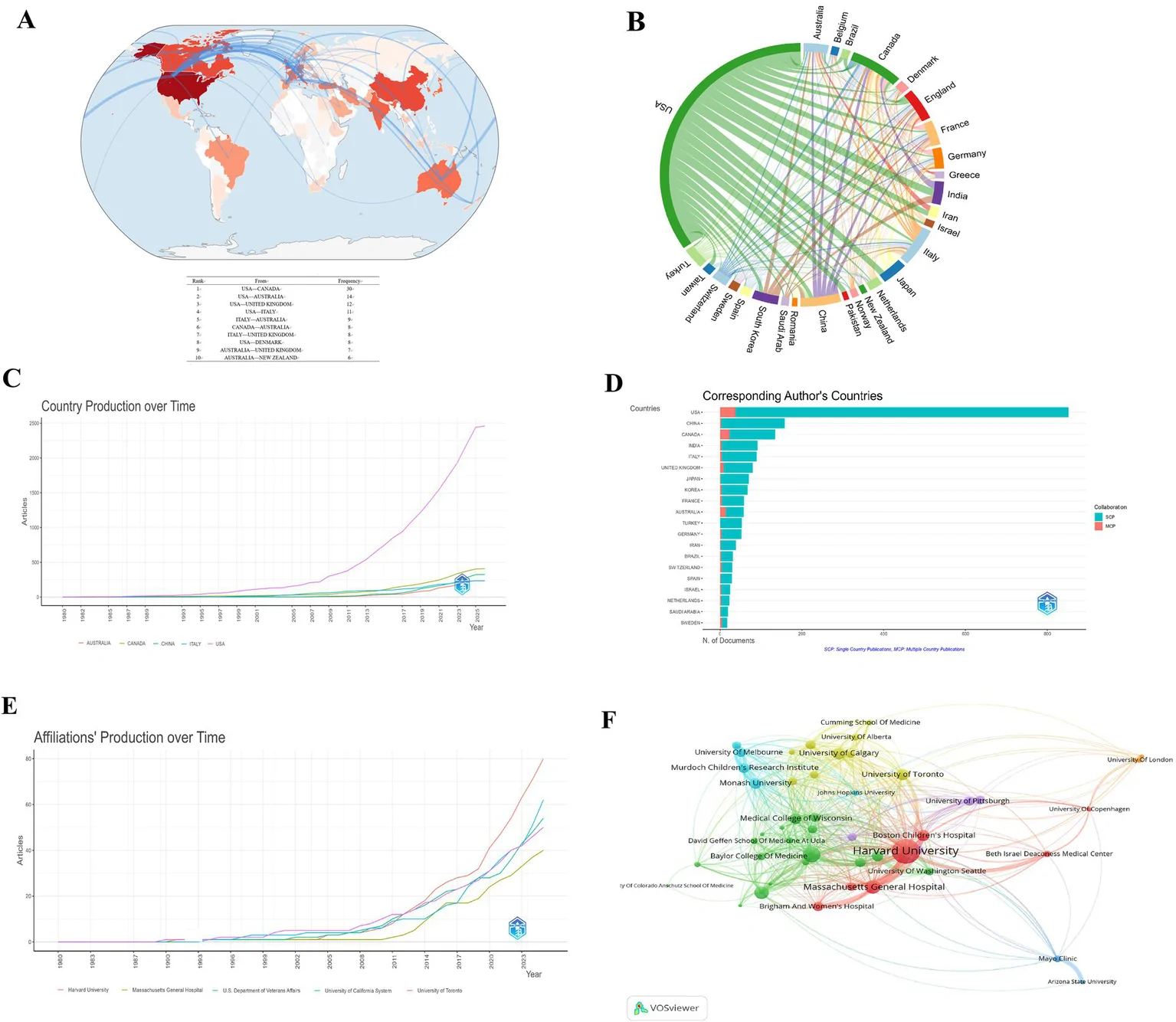
Visualization map of collaboration network and scientific production for countries/regions and institutions. **(A)** Distribution map of collaboration between countries/regions. **(B)** Chord diagram of collaboration distribution between countries/regions. **(C)** Country production over time for the top five productive countries. **(D)** Top 20 countries by number of corresponding authors. **(E)** Temporal trends in scientific production of the leading affiliations. **(F)** Network visualization of collaborations among institutions.

The global collaboration landscape is shown in Figure 4. The United States occupied the central position in the international collaboration network and showed strong collaborative links with Canada, Australia, and the United Kingdom. In terms of publication output, the United States was the most productive country, followed by Canada, China, Italy, and Australia.

At the institutional level, Harvard University ranked first after affiliation name standardization, with 103 articles. It was followed by the University of California System (73 articles), the U. S. Department of Veterans Affairs (65 articles), Mayo Clinic (52 articles), and the University of Toronto (52 articles). Other productive affiliations included Massachusetts General Hospital, the University of Calgary, the University of Pittsburgh, the University of Washington, and the Pennsylvania Commonwealth System of Higher Education (Supplementary Table S5; Figure 4). The institutional collaboration network further showed that high output institutions were mainly concentrated in North America, especially in the United States and Canada.

### Author analysis

Supplementary Figure S1 and Supplementary Table S6 show the analysis of influential authors. Schwedt TJ ranked first by H-index, with an H-index of 14, 25 publications, and 547 citations. Chong CD ranked second, with an H-index of 12, 18 publications, and 454 citations, followed by Iverson GL, with an H-index of 11, 13 publications, and 1,207 citations.

The author collaboration network suggested that several research groups have contributed continuously to this field. However, the overall collaboration structure remained relatively dispersed, with limited connections among some author clusters.

### Citation analysis of documents and references

As shown in Supplementary Table S7, the most locally cited document was the study by Haydel et al. (2000), published in *The New England Journal of Medicine* in 2000 (LC = 47, GC = 785). This was followed by Palchak et al. (2003), published in *Annals of Emergency Medicine* in 2003 (LC = 17, GC = 174), Quayle et al. (1997), published in *Pediatrics* in 1997 (LC = 13, GC = 156), and Ingebrigtsen et al. (1998), published in *Journal of Neurology* in 1998 (LC = 12, GC = 159).

The top 10 cited references ranked by LC are shown in Supplementary Table S8. The most frequently cited reference within the included dataset was the study by Haydel et al. (2000), published in *The New England Journal of Medicine* in 2000 (LC = 139). This was followed by Lucas et al. (2014), published in *Cephalalgia* in 2014 (LC = 67), Stiell et al. (2001), published in *Lancet* in 2001 (LC = 63), Headache Classification Committee of the International Headache Society (IHS) (2018), published in *Cephalalgia* in 2018 (LC = 61), and Kuppermann et al. (2009), published in *Lancet* in 2009 (LC = 58). These highly cited publications represent two related yet distinct clinical research trajectories. One primarily focused on acute TBI assessment, particularly CT-based lesion detection and risk stratification, whereas the other focused more directly on PTH classification, persistent post-traumatic symptoms, and the use of advanced neuroimaging.

### Keyword co-occurrence, burst keyword, and thematic trends

**Figure 5:**
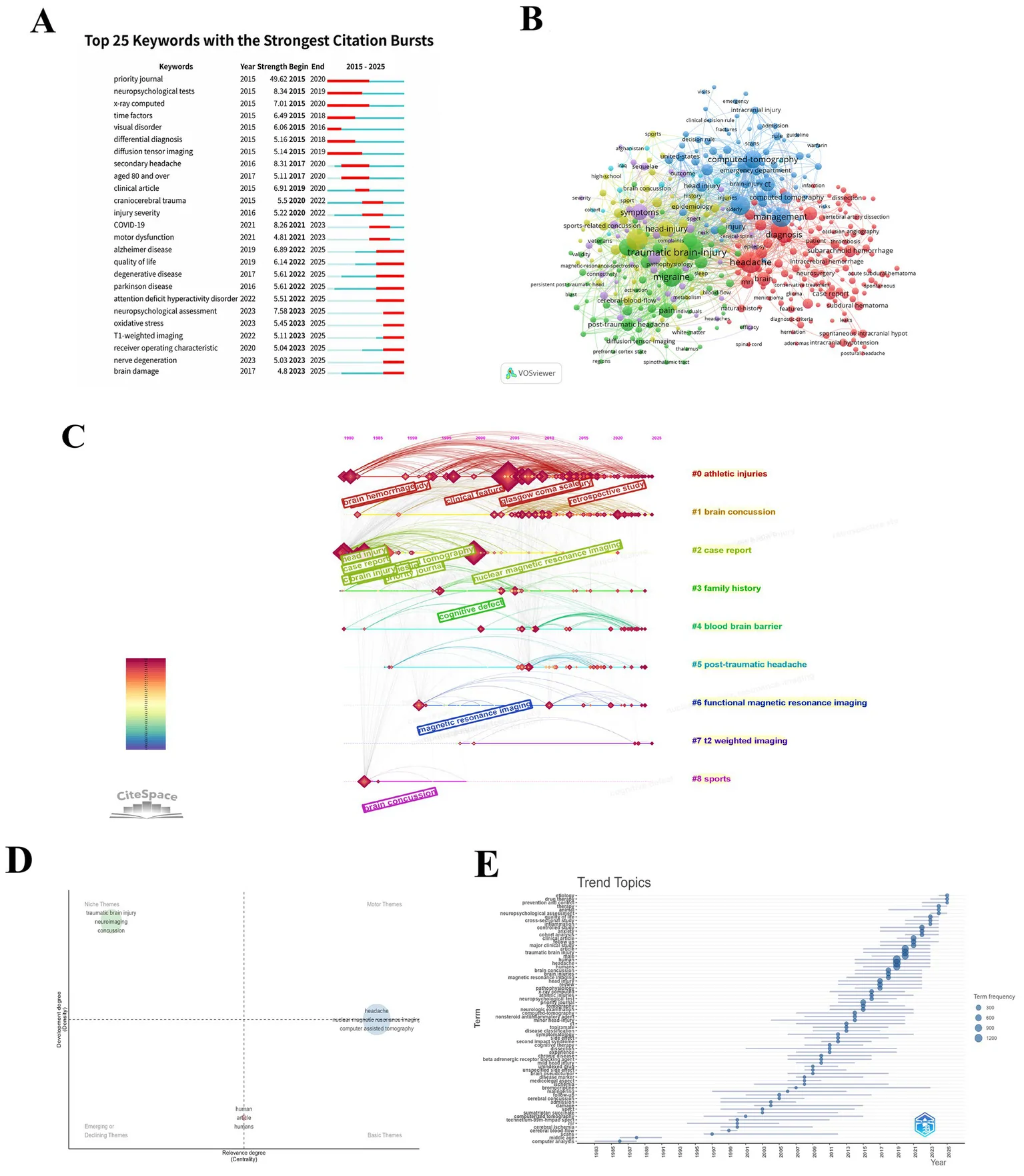
Visualization map of keyword and thematic trends. **(A)** Top 25 keywords with the strongest citation bursts. **(B)** Keyword co-occurrence network. **(C)** Timeline visualization of keyword clusters. **(D)** Thematic map based on centrality and density. **(E)** Trend topics in neuroimaging research related to PTH after TBI over time. TBI, traumatic brain injury. PTH, post-traumatic headache.

Figure 5 shows the visualization map of keyword and thematic trend. In the co-occurrence network, the larger nodes were mainly related to traumatic brain injury, computed tomography, headache, head injury, brain concussion, diagnosis, and magnetic resonance imaging. The top 10 high-frequency keywords were traumatic brain injury (203 occurrences), computed tomography (92 occurrences), management (78 occurrences), head injury (76 occurrences), children (63 occurrences), mild traumatic brain injury (63 occurrences), diagnosis (50 occurrences), symptoms (48 occurrences), risk (42 occurrences), and injury (39 occurrences). Although traumatic brain injury, head injury, and injury appeared as separate terms because of differences in keyword use and database indexing, they all pointed to the central role of TBI-related injury assessment.

Burst keyword analysis identified that early burst terms included neuropsychological tests, x-ray computed, time factors, visual disorder, differential diagnosis, diffusion tensor imaging, secondary headache, aged 80 and over, clinical article, and craniocerebral trauma. More recent burst terms included Alzheimer disease, quality of life, degenerative disease, Parkinson disease, attention deficit hyperactivity disorder, neuropsychological assessment, oxidative stress, T1-weighted imaging, receiver operating characteristic, nerve degeneration, and brain damage.

The timeline visualization identified several clusters, including athletic injuries, brain concussion, case report, family history, blood brain barrier, post-traumatic headache, functional magnetic resonance imaging, T2-weighted imaging, and sports (Figure 5C). In the thematic map, traumatic brain injury, neuroimaging, and concussion were located in the niche themes quadrant, suggesting a relatively specialized but internally developed topic group. Headache, nuclear magnetic resonance imaging, and computer-assisted tomography were positioned closer to the central area of the map, indicating their broad relevance to the field (Figure 5D). Trend-topic analysis showed that earlier terms included computer analysis, cerebral blood flow, child, and single-photon emission computed tomography, whereas more recent terms included traumatic brain injury, headache, brain concussion, neuropsychological assessment, quality of life, therapy, prevention and control, and etiology (Figure 5E). Collectively, these temporal trends suggest that early literature was more strongly characterized by acute brain injury assessment and imaging-based evaluation, while recent research shows greater emphasis on persistent symptoms, advanced neuroimaging, functional prognosis, and longer-term management after TBI.

### Document-type sensitivity analysis

The sensitivity analysis including only original research publications showed that the principal bibliometric analysis remained highly robust after excluding reviews. Annual publication trends between the combined corpus and the subset containing only original research publications were nearly identical (Pearson r = 0.998). The Top 15 journals showed substantial overlap (Jaccard similarity = 0.77) and highly consistent rankings (rho = 0.957). Similarly, the Top 15 keywords remained highly stable (Jaccard similarity = 0.88; rho = 0.917). However, reviews had higher citation counts than original research publications, with mean total citations of 41.8 versus 19.7 and median total citations of 13 versus 6. These findings indicate that temporal, journal, and thematic patterns were robust after excluding reviews, while citation analysis was more sensitive to publication type (Supplementary Table S9).

### Multi-database validation

Supplementary Figure S2 presents the multi-database validation results. Annual publication trends were highly consistent across databases. The Pearson correlation coefficient was 0.981 between WoSCC and Scopus and 0.958 between WoSCC and PubMed, with both correlations reaching statistical significance.

In contrast, the agreement of keyword- and journal-level results was lower. For the top 15 standardized keywords, the Jaccard similarity was 0.071 between WoSCC and Scopus, 0.200 between WoSCC and PubMed, and 0.500 between Scopus and PubMed. Spearman’s rank correlation analysis also showed inconsistent keyword rankings across databases, with negative correlations for WoSCC versus Scopus (rho = −0.535, *p* = 0.003) and WoSCC versus PubMed (rho = −0.482, *p* = 0.015), and a weak non-significant correlation for Scopus versus PubMed (rho = 0.337, *p* = 0.147).

For core journals, the Jaccard similarity values were 0.35 for WoSCC versus Scopus, 0.24 for WoSCC versus PubMed, and 0.25 for Scopus versus PubMed. Spearman’s rank correlation analysis showed weak and non-significant correlations in core journal rankings across all database pairs. These results indicate that annual publication trends were relatively stable across databases, whereas keyword and source analyses were more affected by database coverage, indexing practices, and metadata completeness.

## Discussion

This bibliometric analysis provides a systematic overview of neuroimaging research related to PTH after TBI. By integrating WoSCC and Scopus records and using PubMed for supplementary validation, we identified publication trends, core journals, influential contributors, collaboration networks, citation structures, keyword evolution, and emerging topics in this interdisciplinary field. The results suggest that the included publications reflect several interconnected research contexts, ranging from acute CT-based assessment of head injury and intracranial lesions to studies using advanced neuroimaging techniques to characterize structural and functional alterations associated with persistent PTH and other persistent post-traumatic symptoms. The relevance validation of randomly selected records further supported this pattern. Overall, recent research has increasingly focused on using advanced MRI techniques to investigate persistent symptoms, neural mechanisms, prognosis, and rehabilitation-oriented management (Christensen et al., 2025a, 2025b; Rau et al., 2018; Ofoghi et al., 2020).

This transition is clinically meaningful. PTH is one of the most common sequelae after TBI, but its diagnosis and evaluation remain difficult because its clinical manifestations are heterogeneous and may overlap with migraine or tension headache phenotypes [Ashina et al., 2019; Headache Classification Committee of the International Headache Society (IHS), 2018; Ashina et al., 2021). In patients with mild TBI, conventional CT or routine structural imaging may be normal despite persistent headache, dizziness, cognitive complaints, sleep disturbance, emotional symptoms, and reduced daily functioning (Eagle et al., 2025; Rau et al., 2018). This mismatch between symptom burden and conventional imaging findings helps explain the bibliometric shift from CT-centered injury detection toward advanced neuroimaging such as diffusion imaging, functional MRI, MRS, perfusion imaging, and connectivity-based methods (Christensen et al., 2025a, 2025b; Ofoghi et al., 2020).

The source distribution further highlights the interdisciplinary nature of this topic. Neurotrauma, emergency medicine, and neurosurgery contributed the acute injury-assessment framework, including CT indications and risk stratification after minor head trauma (Haydel et al., 2000; Stiell et al., 2001; Kuppermann et al., 2009). Headache medicine and neurology provided the diagnostic framework for PTH classification, headache phenotyping, and post-concussion symptom assessment [Ashina et al., 2019; Headache Classification Committee of the International Headache Society (IHS), 2018; Ashina et al., 2021]. Neuroradiology and neuroimaging research expanded the field toward structural MRI, functional MRI, diffusion imaging, and network-level mechanisms (Christensen et al., 2025a, 2025b; Rau et al., 2018; Ofoghi et al., 2020). Rehabilitation medicine and neuropsychology further shifted the topic toward persistent symptoms, quality of life, functional recovery, and long-term management. This disciplinary convergence explains why the literature contains both CT-based studies of acute head injury and MRI-based studies of persistent post-traumatic symptoms.

An important task for the field is to bridge the gaps between the partially parallel research frameworks that have developed across neurotrauma, headache medicine, neuroradiology, rehabilitation, and neuropsychology. Acute injury imaging has primarily emphasized lesion detection and risk stratification, whereas headache medicine has focused on clinical phenotyping and diagnostic classification. Moreover, advanced neuroimaging has increasingly addressed structural and functional mechanisms of persistent symptoms. These perspectives are complementary rather than competing. To achieve greater integration, it is necessary to use shared ICHD-3-based PTH definitions and phenotyping, harmonized imaging protocols and prognostic assessment frameworks, and closer multidisciplinary collaboration, so that acute injury characteristics, persistent headache trajectories, neuroimaging findings, and functional prognosis can be interpreted within a common longitudinal framework.

The geographic and institutional results showed a clear concentration of research activity in North America. The United States occupied the central position in the international collaboration network, and several leading institutions were located in the United States and Canada. This pattern may reflect established TBI cohorts, access to advanced imaging platforms, and long-standing collaboration among trauma, neurology, rehabilitation, and imaging groups. However, the institutional collaboration network remained relatively dispersed, indicating that broader multicenter collaboration is still needed, especially for longitudinal studies requiring harmonized imaging protocols, standardized clinical phenotyping, and larger patient samples.

The author analysis also supports the mixed intellectual origins of the field. Productive authors represented partially overlapping communities, including PTH, concussion, pediatric head injury, neuroimaging, and clinical outcome assessment. Although several active author clusters have contributed continuously to this field, the overall collaboration structure has not yet formed a highly integrated network. Future studies would benefit from closer collaboration among headache specialists, neurotrauma teams, neuroradiologists, rehabilitation researchers, neuropsychologists, and data-science groups.

The highly cited documents and references clarify how this research area was formed. Early influential studies mainly focused on CT indications, acute head injury assessment, and pediatric risk stratification after minor head trauma (Haydel et al., 2000; Stiell et al., 2001; Kuppermann et al., 2009). These studies shaped the clinical framework for determining when neuroimaging is needed in emergency settings. In contrast, another group of influential references focused on PTH epidemiology, diagnostic classification, clinical characteristics, and post-concussion symptoms [Ashina et al., 2019; Lucas et al., 2014; Headache Classification Committee of the International Headache Society (IHS), 2018; Ashina et al., 2021]. Therefore, the corpus of neuroimaging research related to PTH after TBI in our bibliometric analysis reflects two related but distinct research trajectories, acute TBI imaging centered on intracranial injury detection and risk stratification, and PTH-related research increasingly focused on persistent symptoms and advanced neuroimaging. For patients with mild TBI and persistent PTH, the latter component is particularly important because symptoms may not be explained by visible lesions alone.

The keyword and thematic results are consistent with this interpretation. High-frequency terms such as computed tomography, management, head injury, diagnosis, and children indicate that imaging-based injury assessment remains central. Meanwhile, recent burst terms and trend topics, including neuropsychological assessment, quality of life, T1-weighted imaging, receiver operating characteristic, nerve degeneration, therapy, and etiology, suggest growing attention to longer-term clinical consequences and imaging markers. Thus, although acute injury assessment remains an important component of the included publications, recent research indicates growing interest in whether advanced neuroimaging can characterize persistent symptoms, predict recovery, and support individualized follow-up.

Recent advanced neuroimaging studies illustrate the type of mechanism-oriented research that has become increasingly visible in these literatures. Studies have reported structural differences between persistent PTH and migraine, as well as reduced cortical thickness in patients with persistent PTH (Schwedt et al., 2017; Chong et al., 2018). Functional imaging studies have suggested that altered connectivity involving pain-processing and default-mode networks may be associated with persistent PTH or the development of chronic pain after mild TBI (Li et al., 2021; Niu et al., 2019). These selected studies exemplify the use of advanced neuroimaging to investigate brain networks and neural mechanisms in persistent PTH, although the specificity and clinical utility of these imaging findings require further validation.

From a clinical and translational perspective, future studies should move beyond isolated cross-sectional imaging findings. Multicenter longitudinal cohorts are needed to clarify how neuroimaging alterations evolve from the acute phase to persistent PTH. Standardized PTH definitions, harmonized imaging protocols, and consistent symptom assessments are essential for improving comparability across studies (Ashina et al., 2021; Ashina et al., 2024). Integrating neuroimaging with symptom trajectories, neuropsychological assessment, and blood biomarkers may help identify patients at risk of persistent headache, predict recovery, and guide individualized follow-up (Eagle et al., 2025; Richter et al., 2024). This direction is particularly important for mild TBI patients with normal CT findings, as advanced imaging and biomarkers may provide additional information about their rehabilitation trajectory (Richter et al., 2024).

**Figure 6:**
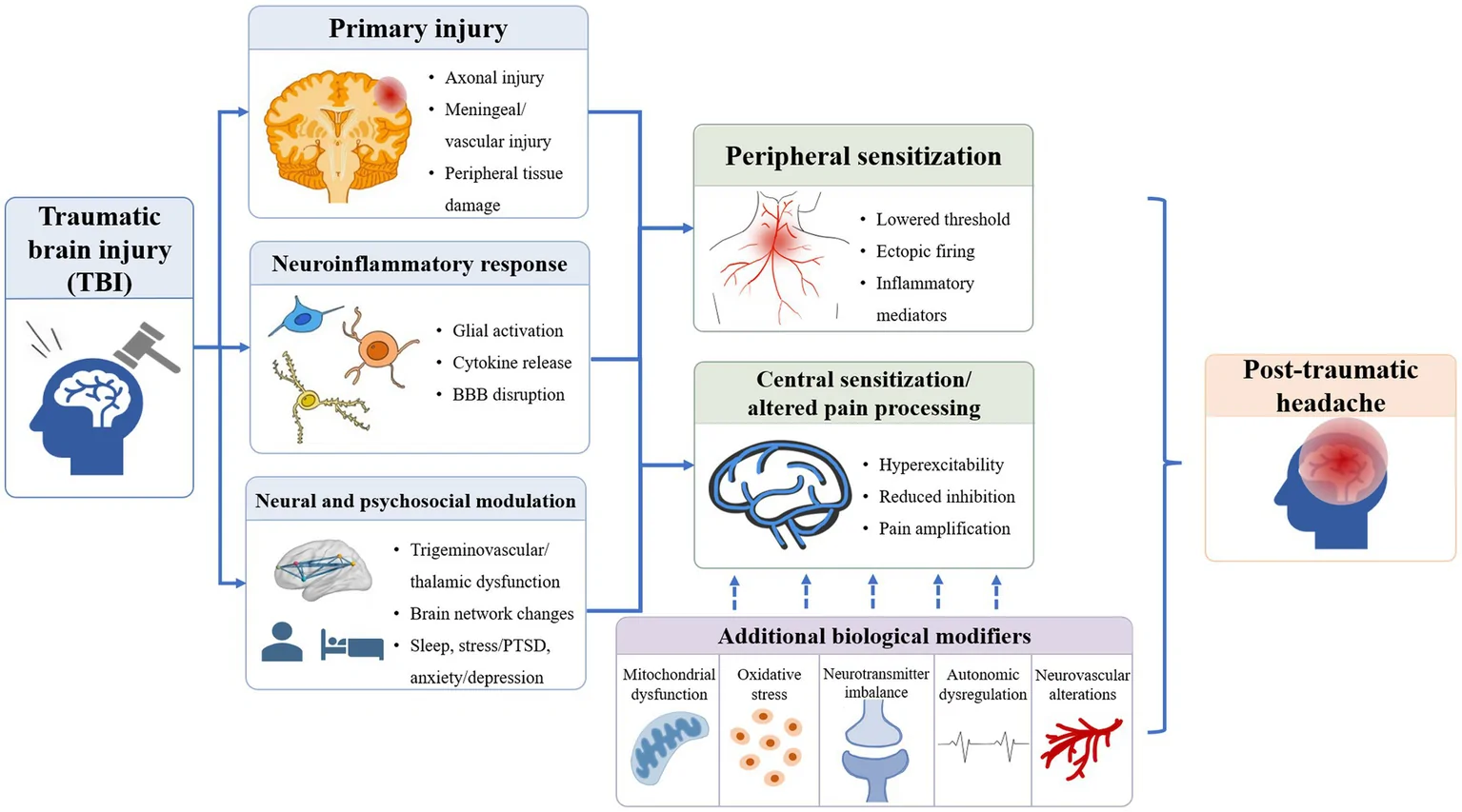
Proposed mechanisms of persistent PTH after TBI. TBI may contribute to persistent PTH through multiple interacting processes. Primary injury, neuroinflammatory response, and neural/psychosocial modulation promote peripheral sensitization and central sensitization/altered pain processing. These changes may be further influenced by additional biological modifiers, including mitochondrial dysfunction, oxidative stress, neurotransmitter imbalance, autonomic dysregulation, and neurovascular alterations.

The proposed mechanism map in Figure 6 provides a useful framework for interpreting these trends. Persistent PTH after TBI may involve primary injury, neuroinflammatory responses, peripheral sensitization, central sensitization, altered pain processing, and neural or psychosocial modulation (Ashina et al., 2019; Ashina et al., 2021). These mechanisms may also be influenced by oxidative stress, mitochondrial dysfunction, neurovascular alterations, neurotransmitter imbalance, and autonomic dysregulation (Ji et al., 2018; Fesharaki-Zadeh, 2022). This framework is consistent with the increasing visibility of terms related to neuropsychological assessment, quality of life, brain damage, nerve degeneration, and imaging-related markers. Therefore, future neuroimaging studies should examine not only structural lesions, but also functional connectivity, pain-processing networks, neurovascular regulation, and multimodal clinical–imaging phenotypes.

Several populations deserve particular attention. Children appeared among the high-frequency keywords, which is consistent with the clinical importance of pediatric head injury and the need to balance diagnostic yield against radiation exposure and developmental vulnerability (Kuppermann et al., 2009; Lumba-Brown et al., 2018). Athletes and patients with sports-related concussion represent another important group because repeated mild TBI may influence symptom persistence, recovery monitoring, and return-to-play decisions (Patricios et al., 2023). Veterans are also clinically relevant because blast exposure, repeated mild TBI, neuropsychiatric symptoms, and persistent post-traumatic symptoms may interact in complex ways (Kim et al., 2023). These populations may share some mechanisms, but their risk factors, symptom profiles, imaging characteristics, and recovery trajectories are unlikely to be identical. Therefore, future studies should develop population-specific imaging and clinical stratification strategies.

The multi-database validation adds methodological value to this study. Annual publication trends were highly consistent across WoSCC, Scopus, and PubMed, supporting the robustness of the macro-level growth pattern. In contrast, keyword and core journal results showed lower agreement across databases. This difference is understandable because annual output is relatively stable, whereas keyword networks and source rankings are more sensitive to database coverage, indexing rules, vocabulary, and metadata completeness (Mongeon and Paul-Hus, 2016; Falagas et al., 2008; Gusenbauer and Haddaway, 2020). For interdisciplinary fields such as our topic, reliance on a single database may overemphasize certain disciplinary communities while underrepresenting others. Hence, multi-database integration and validation can provide a more balanced view of research development (Donthu et al., 2021; Montazeri et al., 2023).

This study has several limitations. First, although multiple databases were included, only original publications and reviews in English were analyzed, which may have introduced language and publication-type bias. Second, citation-based indicators are influenced by publication time and may favor older studies, whereas recently published but potentially important studies may be underestimated because of citation lag. Third, bibliometric analysis is descriptive and cannot directly assess the methodological quality, clinical validity, or causal impact of individual studies. Finally, the interdisciplinary scope of this topic should be considered when interpreting the findings. Neuroimaging research related to PTH after TBI has encompassed the overlapping fields of neurosurgery, neuroradiology, headache medicine and rehabilitation, meaning this bibliometric analysis includes research with varied degrees of direct relevance to this field. We addressed this issue analytically through sample relevance validation. Therefore, the results should be interpreted as a broad mapping of neuroimaging research related to PTH after TBI.

## Conclusion

In this broad multi-database bibliometric dataset linking TBI, PTH and neuroimaging, we identified a long-standing knowledge base in acute TBI imaging and related symptom assessment, alongside an increasingly visible research trajectory focused more directly on persistent PTH, advanced neuroimaging, underlying mechanisms, prognosis, and rehabilitation-oriented management. Future research should prioritize multicenter longitudinal cohorts, standardized imaging protocols, harmonized clinical definitions of PTH, and multimodal models integrating neuroimaging with symptom profiles, neuropsychological measures, and biological markers. Such efforts may help clarify the mechanisms of persistent PTH and improve risk stratification, follow-up planning, and individualized management after TBI.

## Data Availability

The raw data supporting the conclusions of this article will be made available by the authors, without undue reservation.

## References

[ref1] Aria M. Cuccurullo C. (2017). Bibliometrix: an R-tool for comprehensive science mapping analysis. J. Informetr. 11, 959–975. doi: 10.1016/j.joi.2017.08.007

[ref2] Ashina H. Diener H.-C. Tassorelli C. Scher A. I. Lipton R. B. Pozo-Rosich P. . (2024). Guidelines of the international headache society for controlled trials of pharmacological preventive treatment for persistent post-traumatic headache attributed to mild traumatic brain injury. Cephalalgia 44:03331024241234068. doi: 10.1177/03331024241234068 38518177

[ref3] Ashina H. Dodick D. W. Barber J. Temkin N. R. Chong C. D. Adler J. S. . (2023). Prevalence of and risk factors for post-traumatic headache in civilian patients after mild traumatic brain injury: a TRACK-TBI study. Mayo Clin. Proc. 98, 1515–1526. doi: 10.1016/j.mayocp.2023.02.026, 37480909

[ref4] Ashina H. Eigenbrodt A. K. Seifert T. Sinclair A. J. Scher A. I. Schytz H. W. . (2021). Post-traumatic headache attributed to traumatic brain injury: classification, clinical characteristics, and treatment. Lancet Neurol. 20, 460–469. doi: 10.1016/S1474-4422(21)00094-6, 34022171

[ref5] Ashina H. Porreca F. Anderson T. Amin F. M. Ashina M. Schytz H. W. . (2019). Post-traumatic headache: epidemiology and pathophysiological insights. Nat. Rev. Neurol. 15, 607–617. doi: 10.1038/s41582-019-0243-8, 31527806

[ref6] Chen C. (2006). CiteSpace II: detecting and visualizing emerging trends and transient patterns in scientific literature. J. Am. Soc. Inf. Sci. Technol. 57, 359–377. doi: 10.1002/asi.20317

[ref7] Chong C. D. Berisha V. Chiang C. C. Ross K. Schwedt T. J. (2018). Less cortical thickness in patients with persistent post-traumatic headache compared with healthy controls: an MRI study. Headache 58, 53–61. doi: 10.1111/head.13223, 29139130 PMC5750114

[ref8] Christensen R. H. Al-Khazali H. M. Iljazi A. Szabo E. Ashina H. (2025a). Functional magnetic resonance imaging of post-traumatic headache: a systematic review. Curr. Pain Headache Rep. 29:27. doi: 10.1007/s11916-024-01351-2, 39812946

[ref9] Christensen R. H. Al-Khazali H. M. Iljazi A. Szabo E. Ashina H. (2025b). Structural magnetic resonance imaging of post-traumatic headache: a systematic review. Curr. Pain Headache Rep. 29:20. doi: 10.1007/s11916-024-01341-4, 39775377

[ref10] Dewan M. C. Rattani A. Gupta S. Baticulon R. E. Hung Y. C. Punchak M. . (2019). Estimating the global incidence of traumatic brain injury. J. Neurosurg. 130, 1080–1097. doi: 10.3171/2017.10.JNS17352, 29701556

[ref11] Donthu N. Kumar S. Mukherjee D. Pandey N. Lim W. M. (2021). How to conduct a bibliometric analysis: an overview and guidelines. J. Bus. Res. 133, 285–296. doi: 10.1016/j.jbusres.2021.04.070

[ref12] Du Q. Li Q. Liao G. Li J. Ye P. Zhang Q. . (2023). Emerging trends and focus of research on the relationship between traumatic brain injury and gut microbiota: a visualized study. Front. Microbiol. 14:1278438. doi: 10.3389/fmicb.2023.1278438, 38029105 PMC10654752

[ref13] Eagle S. R. Temkin N. Barber J. K. McCrea M. Giacino J. T. Okonkwo D. . (2025). Association of Subacute Mild Traumatic Brain Injury Symptoms with Long-Term Persistent Symptoms, functional limitations, and quality of life. Neurology 104:e213427. doi: 10.1212/WNL.0000000000213427, 40168631 PMC11966525

[ref14] Egghe L. (2006). Theory and practise of the g-index. Scientometrics 69, 131–152. doi: 10.1007/s11192-006-0144-7

[ref15] Falagas M. E. Pitsouni E. I. Malietzis G. A. Pappas G. (2008). Comparison of PubMed, Scopus, web of science, and Google scholar: strengths and weaknesses. FASEB J. 22, 338–342. doi: 10.1096/fj.07-9492LSF, 17884971

[ref16] Fesharaki-Zadeh A. (2022). Oxidative stress in traumatic brain injury. Int. J. Mol. Sci. 23:13000. doi: 10.3390/ijms232113000, 36361792 PMC9657447

[ref17] Gusenbauer M. Haddaway N. R. (2020). Which academic search systems are suitable for systematic reviews or meta-analyses? Evaluating retrieval qualities of Google scholar, PubMed, and 26 other resources. Res. Synth. Methods 11, 181–217. doi: 10.1002/jrsm.1378, 31614060 PMC7079055

[ref18] Haydel M. J. Preston C. A. Mills T. J. Luber S. Blaudeau E. DeBlieux P. M. (2000). Indications for computed tomography in patients with minor head injury. N. Engl. J. Med. 343, 100–105. doi: 10.1056/nejm200007133430204, 10891517

[ref9001] Headache Classification Committee of the International Headache Society (IHS). (2018). The international classification of headache disorders, 3rd edition. Cephalalgia. 38, 1–211. doi: 10.1177/0333102417738202, 29368949

[ref19] Hirsch J. E. (2005). An index to quantify an individual’s scientific research output. Proc. Natl. Acad. Sci. 102, 16569–16572. doi: 10.1073/pnas.0507655102, 16275915 PMC1283832

[ref20] Ingebrigtsen T. Waterloo K. Marup-Jensen S. Attner E. Romner B. (1998). Quantification of post-concussion symptoms 3 months after minor head injury in 100 consecutive patients. J. Neurol. 245, 609–612. doi: 10.1007/s004150050254, 9758300

[ref21] Ji R. R. Nackley A. Huh Y. Terrando N. Maixner W. (2018). Neuroinflammation and central sensitization in chronic and widespread pain. Anesthesiology 129, 343–366. doi: 10.1097/ALN.0000000000002130, 29462012 PMC6051899

[ref22] Kim S. Ollinger J. Song C. Raiciulescu S. Seenivasan S. Wolfgang A. . (2024). White matter alterations in military service members with remote mild traumatic brain injury. JAMA Netw. Open 7:e248121. doi: 10.1001/jamanetworkopen.2024.8121, 38635266 PMC11161843

[ref23] Kim S. Y. Yeh P. H. Ollinger J. M. Morris H. D. Hood M. N. Ho V. B. . (2023). Military-related mild traumatic brain injury: clinical characteristics, advanced neuroimaging, and molecular mechanisms. Transl. Psychiatry 13:289. doi: 10.1038/s41398-023-02569-1, 37652994 PMC10471788

[ref24] Kuppermann N. Holmes J. F. Dayan P. S. Hoyle J. D. Atabaki S. M. Holubkov R. . (2009). Identification of children at very low risk of clinically-important brain injuries after head trauma: a prospective cohort study. Lancet 374, 1160–1170. doi: 10.1016/S0140-6736(09)61558-0, 19758692

[ref25] Li F. Lu L. Shang S. Chen H. Wang P. Muthaiah V. P. . (2021). Altered static and dynamic functional network connectivity in post-traumatic headache. J. Headache Pain 22:137. doi: 10.1186/s10194-021-01348-x, 34773973 PMC8590227

[ref26] Lucas S. Hoffman J. M. Bell K. R. Dikmen S. (2014). A prospective study of prevalence and characterization of headache following mild traumatic brain injury. Cephalalgia 34, 93–102. doi: 10.1177/0333102413499645, 23921798

[ref27] Lumba-Brown A. Yeates K. O. Sarmiento K. Breiding M. J. Haegerich T. M. Gioia G. A. . (2018). Centers for Disease Control and Prevention guideline on the diagnosis and Management of Mild Traumatic Brain Injury among Children. JAMA Pediatr. 172:e182853. doi: 10.1001/jamapediatrics.2018.2853, 30193284 PMC7006878

[ref28] Maas A. I. R. Menon D. K. Manley G. T. Abrams M. Åkerlund C. Andelic N. . (2022). Traumatic brain injury: progress and challenges in prevention, clinical care, and research. Lancet Neurol. 21, 1004–1060. doi: 10.1016/S1474-4422(22)00309-X, 36183712 PMC10427240

[ref29] McGeown J. P. Pedersen M. Mito R. Theadom A. Maller J. J. Condron P. . (2026). Neuroimaging correlates of symptom burden and functional recovery following mild traumatic brain injury: a systematic review. Neuroimage Clin. 49:103910. doi: 10.1016/j.nicl.2025.103910 PMC12811548 41496379

[ref30] Mojgani P. Jalali M. Keramatfar A. (2022). Bibliometric study of traumatic brain injury rehabilitation. Neuropsychol. Rehabil. 32, 51–68. doi: 10.1080/09602011.2020.1796714, 32744132

[ref31] Mongeon P. Paul-Hus A. (2016). The journal coverage of web of science and Scopus: a comparative analysis. Scientometrics 106, 213–228. doi: 10.1007/s11192-015-1765-5

[ref32] Montazeri A. Mohammadi S. M Hesari P. Ghaemi M. Riazi H. Sheikhi-Mobarakeh Z. (2023). Preliminary guideline for reporting bibliometric reviews of the biomedical literature (BIBLIO): a minimum requirements. Syst. Rev. 12:239. doi: 10.1186/s13643-023-02410-2, 38102710 PMC10722750

[ref33] Niu X. Bai L. Sun Y. Wang S. Cao J. Sun C. . (2019). Disruption of periaqueductal grey-default mode network functional connectivity predicts persistent post-traumatic headache in mild traumatic brain injury. J. Neurol. Neurosurg. Psychiatry 90, 326–332. doi: 10.1136/jnnp-2018-318886, 30554137

[ref34] Ofoghi Z. Dewey D. Barlow K. M. (2020). A systematic review of structural and functional imaging correlates of headache or pain after mild traumatic brain injury. J. Neurotrauma 37, 907–923. doi: 10.1089/neu.2019.6750, 31822205

[ref36] Palchak M. J. Holmes J. F. Vance C. W. Gelber R. E. Schauer B. A. Harrison M. J. . (2003). A decision rule for identifying children at low risk for brain injuries after blunt head trauma. Ann. Emerg. Med. 42, 492–506. doi: 10.1067/S0196-0644(03)00425-6, 14520320

[ref37] Patricios J. S. Schneider K. J. Dvorak J. Ahmed O. H. Blauwet C. Cantu R. C. . (2023). Consensus statement on concussion in sport: the 6th international conference on concussion in sport-Amsterdam, October 2022. Br. J. Sports Med. 57, 695–711. doi: 10.1136/bjsports-2023-106898, 37316210

[ref38] Polinder S. Cnossen M. C. Real R. G. L. Covic A. Gorbunova A. Voormolen D. C. . (2018). A multidimensional approach to post-concussion symptoms in mild traumatic brain injury. Front. Neurol. 9:1113. doi: 10.3389/fneur.2018.01113, 30619066 PMC6306025

[ref39] Quayle K. S. Jaffe D. M. Kuppermann N. Kaufman B. A. Lee B. C. Park T. S. . (1997). Diagnostic testing for acute head injury in children: when are head computed tomography and skull radiographs indicated? Pediatrics 99, e11–e11. doi: 10.1542/peds.99.5.e11, 9113968

[ref40] Rau J. C. Dumkrieger G. M. Chong C. D. Schwedt T. J. (2018). Imaging post-traumatic headache. Curr. Pain Headache Rep. 22:64. doi: 10.1007/s11916-018-0719-z, 30062548

[ref41] Richter S. Winzeck S. Correia M. M. Czeiter E. Whitehouse D. Kornaropoulos E. N. . (2024). Predicting recovery in patients with mild traumatic brain injury and a normal CT using serum biomarkers and diffusion tensor imaging (CENTER-TBI): an observational cohort study. EClinicalMedicine 75:102751. doi: 10.1016/j.eclinm.2024.102751, 39720677 PMC11667275

[ref42] Sang X. Z. Wang C. Q. Chen W. Rong H. Hou L. J. (2023). An exhaustive analysis of post-traumatic brain injury dementia using bibliometric methodologies. Front. Neurol. 14:1165059. doi: 10.3389/fneur.2023.1165059, 37456644 PMC10345842

[ref43] Schwedt T. J. (2021). Post-traumatic headache due to mild traumatic brain injury: current knowledge and future directions. Cephalalgia 41, 464–471. doi: 10.1177/0333102420970188, 33210546

[ref44] Schwedt T. J. Chong C. D. Peplinski J. Ross K. Berisha V. (2017). Persistent post-traumatic headache vs. migraine: an MRI study demonstrating differences in brain structure. J. Headache Pain 18:87. doi: 10.1186/s10194-017-0796-0, 28831776 PMC5567584

[ref45] Stiell I. G. Wells G. A. Vandemheen K. Clement C. Lesiuk H. Laupacis A. . (2001). The Canadian CT head rule for patients with minor head injury. Lancet 357, 1391–1396. doi: 10.1016/S0140-6736(00)04561-X, 11356436

[ref46] van Eck N. J. Waltman L. (2010). Software survey: VOSviewer, a computer program for bibliometric mapping. Scientometrics 84, 523–538. doi: 10.1007/s11192-009-0146-3, 20585380 PMC2883932

[ref47] Zupic I. Čater T. (2015). Bibliometric methods in management and organization. Organ. Res. Methods 18, 429–472. doi: 10.1177/1094428114562629

