# Supplementary material for: Mapping neuroimaging research related to post-traumatic headache after traumatic brain injury: a multi-database bibliometric analysis
Source: Front Neurosci. 2026 Sep 9;20:1923958. doi: 10.3389/fnins.2026.1923958 (PMC13597782; doi:10.3389/fnins.2026.1923958)
Supplement: Supplementary file 1 [file Supplementary_file_1.docx]

**Table S1** Search strategies for Scopus.

| **Step** | **Search strategy** |
| --- | --- |
| #1 | TITLE-ABS-KEY("Brain Injuries, Traumatic" OR "Brain Injury, Traumatic" OR  "Traumatic Brain Injuries" OR "Trauma*, Brain" OR "Brain Trauma*" OR TBI* OR mTBI* OR "Encephalopathy, Traumatic" OR "Encephalopathies, Traumatic" OR "Traumatic Encephalopathies" OR "Injury, Brain, Traumatic" OR "Traumatic Encephalopathy" OR "Traumatic Brain Injury" OR "Cerebral trauma" OR "Post-traumatic" OR "Post-concussion" OR Concussion* OR Concussive OR "Commotio Cerebri") |
| #2 | TITLE-ABS-KEY( Headache* OR "Secondary Headache" OR "Post-Traumatic Headache" OR "Post Traumatic Headache" OR "Head Pain*" OR "Pain*, Head" OR Cephalalgia* OR "Cranial Pain*" OR Cephalgia* OR Hemicrania) |
| #3 | TITLE-ABS-KEY( imaging OR neuroimaging OR neuroradiology OR "computed tomography" OR CT OR MRI OR DKI OR DWI OR DTI OR PWI OR "arterial spin labeling" OR "arterial spin labelling" OR ASL OR "magnetic resonance spectroscopy" OR MRS OR "positron emission tomography" OR PET OR "single photon emission computed tomography" OR SPECT OR network OR connect* OR ultrasound OR doppler) |
| #4 | #1 AND #2 AND #3 |

**Table S2** Search strategies for PubMed.

| **Step** | **Search strategy** |
| --- | --- |
| #1 | ("Brain Injuries, Traumatic"[Mesh] OR "Brain Concussion"[Mesh] OR "traumatic brain injury"[Title/Abstract] OR "traumatic brain injuries"[Title/Abstract] OR "brain injury, traumatic"[Title/Abstract] OR "brain injuries, traumatic"[Title/Abstract] OR "brain trauma"[Title/Abstract] OR "cerebral trauma"[Title/Abstract] OR post-traumatic[Title/Abstract] OR postconcussion[Title/Abstract] OR "post-concussion"[Title/Abstract] OR concussion*[Title/Abstract] OR concussive[Title/Abstract] OR "commotio cerebri"[Title/Abstract] OR TBI*[Title/Abstract] OR mTBI*[Title/Abstract]) |
| #2 | ("Post-Traumatic Headache"[Mesh] OR "Headache Disorders, Secondary"[Mesh] OR "Headache"[Mesh] OR headache*[Title/Abstract] OR "post-traumatic headache"[Title/Abstract] OR "post traumatic headache"[Title/Abstract] OR  "secondary headache"[Title/Abstract] OR "head pain"[Title/Abstract] OR cephalalgia*[Title/Abstract] OR cephalgia*[Title/Abstract] OR "cranial pain"[Title/Abstract] OR hemicrania[Title/Abstract]) |
| #3 | ("Diagnostic Imaging"[Mesh] OR "Neuroimaging"[Mesh] OR "Tomography, X-Ray Computed"[Mesh] OR "Magnetic Resonance Imaging"[Mesh] OR "Diffusion Magnetic Resonance Imaging"[Mesh] OR "Diffusion Tensor Imaging"[Mesh] OR "Perfusion Magnetic Resonance Imaging"[Mesh] OR "Magnetic Resonance Spectroscopy"[Mesh] OR "Positron-Emission Tomography"[Mesh] OR "Tomography, Emission-Computed, Single-Photon"[Mesh] OR "Ultrasonography, Doppler"[Mesh] OR imaging[Title/Abstract] OR neuroimaging[Title/Abstract] OR neuroradiology[Title/Abstract] OR "computed tomography"[Title/Abstract] OR CT[Title/Abstract] OR MRI[Title/Abstract] OR DWI[Title/Abstract] OR DTI[Title/Abstract] OR DKI[Title/Abstract] OR PWI[Title/Abstract] OR "arterial spin labeling"[Title/Abstract] OR "arterial spin labelling"[Title/Abstract] OR ASL[Title/Abstract] OR "magnetic resonance spectroscopy"[Title/Abstract] OR MRS[Title/Abstract] OR "positron emission tomography"[Title/Abstract] OR PET[Title/Abstract] OR SPECT[Title/Abstract] OR ultrasound[Title/Abstract] OR doppler[Title/Abstract] OR network[Title/Abstract] OR connect*[Title/Abstract]) |
| #4 | #1 AND #2 AND #3 |

**Table S3** Relevance validation of 200 randomly selected records from the principal bibliometric corpus.

| **Classification** | **Definition** | **Records, n (%)** |
| --- | --- | --- |
| Neuroimaging studies related to PTH after TBI | PTH following TBI was the primary clinical focus or research question, and neuroimaging served as a key component of the study. | 17 (8.5%) |
| TBI neuroimaging studies incorporating PTH assessment | Neuroimaging after TBI was the primary research focus, with PTH serving as an important clinical feature, symptomatic measure, predictor or outcome. | 20 (10.0%) |
| Studies with substantial contextual relevance to neuroimaging of PTH after TBI | Studies concerned broader headache, TBI or related clinical topics but included significant analysis, findings, or discussion directly related to the neuroimaging of PTH after TBI | 113 (56.5%) |
| Studies with broader interdisciplinary relevance to neuroimaging of PTH after TBI | The primary research topics were adjacent or intersecting with this area, but the studies contained an explicit and meaningful connection with the neuroimaging of PTH after TBI | 50 (25.0%) |
| PTH, post-traumatic headache. TBI, traumatic brain injury. | | |

**Table S4** The Top 10 Journals Ranked by H-index.

| **Rank** | **Source** | **H-index** | **G-index** | **M-index** | **Publications** | **Citations** |
| --- | --- | --- | --- | --- | --- | --- |
| 1 | Journal of Neurotrauma | 22 | 46 | 0.688 | 51 | 2147 |
| 2 | Headache | 20 | 31 | 0.426 | 46 | 1037 |
| 3 | Cephalalgia | 17 | 31 | 0.459 | 31 | 1017 |
| 4 | Brain Injury | 16 | 35 | 0.41 | 50 | 1308 |
| 5 | Journal of Headache and Pain | 15 | 24 | 0.714 | 27 | 583 |
| 6 | Current Pain and Headache Reports | 14 | 23 | 0.583 | 27 | 557 |
| 7 | Frontiers in Neurology | 14 | 23 | 1.077 | 53 | 605 |
| 8 | World Neurosurgery | 13 | 19 | 0.813 | 61 | 543 |
| 9 | American Journal of Neuroradiology | 11 | 18 | 0.333 | 18 | 406 |
| 10 | Journal of Neurosurgery | 11 | 16 | 0.367 | 16 | 643 |

**Table S5** The Top 10 institutions ranked by publications.

| **Rank** | **Institution** | **Publications** |
| --- | --- | --- |
| 1 | Harvard University | 103 |
| 2 | University of California System | 73 |
| 3 | U.S. Department of Veterans Affairs | 65 |
| 4 | Mayo Clinic | 52 |
| 5 | University of Toronto | 52 |
| 6 | Massachusetts General Hospital | 45 |
| 7 | University of Calgary | 40 |
| 8 | University of Pittsburgh | 37 |
| 9 | University of Washington | 32 |
| 10 | Pennsylvania Commonwealth System of Higher Education | 30 |
|  | | |

**Table S6** The Top 10 Authors Ranked by H-index.

| **Rank** | **Author** | **H-index** | **G-index** | **M-index** | **Publications** | **Citations** |
| --- | --- | --- | --- | --- | --- | --- |
| 1 | Schwedt TJ | 14 | 23 | 1.17 | 25 | 547 |
| 2 | Chong CD | 12 | 18 | 1 | 18 | 454 |
| 3 | Iverson GL | 11 | 13 | 0.61 | 13 | 1207 |
| 4 | Babl FE | 8 | 12 | 0.62 | 12 | 240 |
| 5 | Master CL | 8 | 9 | 0.8 | 9 | 467 |
| 6 | Mayer AR | 8 | 13 | 0.8 | 13 | 423 |
| 7 | Zhang JT | 8 | 13 | 0.44 | 13 | 253 |
| 8 | Berisha V | 7 | 9 | 0.7 | 9 | 293 |
| 9 | Bressan S | 7 | 10 | 0.78 | 10 | 152 |
| 10 | Giza CC | 7 | 11 | 0.37 | 11 | 619 |

**Table S7** The Top 10 documents ranked by local citations.

| **Rank** | **Document** | **DOI** | **LC** | **GC** | **LC/GC Ratio (%)** |
| --- | --- | --- | --- | --- | --- |
| 1 | Haydel MJ, 2000, The New England Journal of Medicine | 10.1056/NEJM200007133430204 | 47 | 785 | 5.99 |
| 2 | Palchak MJ, 2003, Annals of Emergency Medicine | 10.1067/S0196-0644(03)00425-6 | 17 | 174 | 9.77 |
| 3 | Quayle KS, 1997, Pediatrics | 10.1542/peds.99.5.e11 | 13 | 156 | 8.33 |
| 4 | Ingebrigtsen T, 1998, Journal of Neurology | 10.1007/s004150050254 | 12 | 159 | 7.55 |
| 5 | Ibanez J, 2004, Journal of Neurosurgery | 10.3171/jns.2004.100.5.0825 | 11 | 117 | 9.40 |
| 6 | Dietrich AM, 1993, Annals of Emergency Medicine | 10.1016/S0196-0644(05)81254-5 | 10 | 131 | 7.63 |
| 7 | Haydel MJ, 2003, Annals of Emergency Medicine | 10.1067/S0196-0644(03)00512-2 | 10 | 85 | 11.76 |
| 8 | Iverson GL, 2005, Current Opinion in Psychiatry | 10.1097/01.yco.0000165601.29047.ae | 10 | 510 | 1.96 |
| 9 | Murshid WR, 1998, Acta Neurochirurgica | 10.1007/s007010050058 | 8 | 38 | 21.05 |
| 10 | Abdel-Dayem HM, 1998, Clinical Nuclear Medicine | 10.1097/00003072-199805000-00009 | 8 | 126 | 6.35 |

LC, Local Citations. GC, Global Citations.

**Table S8** The Top 10 references by local citations.

| **Rank** | **References** | **DOI** | **LC** |
| --- | --- | --- | --- |
| 1 | Haydel MJ, 2000, The New England Journal of Medicine | 10.1056/NEJM200007133430204 | 139 |
| 2 | Lucas S, 2014, Cephalalgia | 10.1177/0333102413499645 | 67 |
| 3 | Stiell IG, 2001, Lancet | 10.1016/S0140-6736(00)04561-X | 63 |
| 4 | Olesen J, 2018, Cephalalgia | 10.1177/0333102417738202 | 61 |
| 5 | Kuppermann N, 2009, Lancet | 10.1016/S0140-6736(09)61558-0 | 58 |
| 6 | Ashina H, 2019, Nature Reviews Neurology | 10.1038/S41582-019-0243-8 | 57 |
| 7 | Teasdale G, 1974, Lancet | 10.1016/S0140-6736(74)91639-0 | 55 |
| 8 | Cassidy JD, 2004, Journal of Rehabilitation Medicine | 10.1080/16501960410023732 | 53 |
| 9 | McCrory P, 2017, British Journal of Sports Medicine | 10.1136/BJSPORTS-2017-097699 | 51 |
| 10 | King NS, 1995, Journal of Neurology | 10.1007/BF00868811 | 48 |

LC, Local Citations.

**Table S9** Document-type composition and sensitivity analysis of the principal bibliometric corpus.

| **Metric** | **Value** | **Interpretation** |
| --- | --- | --- |
| Current principal corpus | 2519 | WoSCC + Scopus |
| Original research publications | 2008 | 79.7% of principal corpus |
| Reviews | 511 | 20.3% of principal corpus |
| Annual output correlation: original + review vs original | 0.998 | Nearly identical temporal pattern |
| Top 15 journals | | |
| Jaccard | 0.765 | High overlap |
| Rank rho | 0.957 | 13/15 top journals overlapped |
| Top 15 keywords | | |
| Jaccard | 0.875 | High overlap |
| Rank rho | 0.917 | 14/15 top keywords overlapped |
| Mean total citations | | |
| Original research publications | 19.7 |  |
| Reviews | 41.8 |  |
| Median total citations | | |
| Original research publications | 6 |  |
| Reviews | 13 |  |

**Figure S1.** Visualization map of influential authors and their collaborative characteristics. **(A)** Local impact of authors by H-index. **(B)** Annual publication trend of the top 10 authors (each point represents an author’s publications at a particular point in time, the size of the point represents the number of publications, and the color shade of the point represents the number of citations). **(C)** Author collaboration network.

**Figure S2.** The results of multi-database validation. **(A)** Correlation analysis of annual publication trends in different databases. **(B)** Venn diagrams of the top 15 keywords. **(C)** Venn diagrams of core journals.
